# Editorial

**Published:** 1904-06-15

**Authors:** 


### EDITORIAL.

"Too Much Johnson."—A report of the proceedings of the Illinois State Dental Society, as published in the May *American Journal*, states that Dr. C. N. Johnson, the accomplished editor of the *Review*, was elected, both President and Treasurer of that Society. We applaud the good taste of the Society in its selection of Dr. Johnson as its President, at the same time, we wonder why a great Society with more than three hundred members could not relieve him of the responsibility and care of an office which requires only integrity and a little business ability for its satisfactory conduct. It is often said that it is only the capable and busy man who is wanted in responsible positions. This may be a good business proposition in general, but it always seems unreasonable that a man should be asked to do things just because he can do them well. In the development of our profession too little thought has been given to conserving our resources, which are in most cases men rather than money. If we were a millionaire, such a man as Dr. W. D. Miller should not be filling teeth for a living, neither should Dr. G. V. Black be wearing out his life with the details of management of a dental college. Such men can not be developed at will, and it is doubtful if we shall again know their equals. How much richer we should be had we taken thought and given these men the chance to do the work we so much need?

Is there a Call for Specialists in Dentistry?—In a communication to the editor of the *Items of Interest*, Dr. Geo. E. Hunt makes the statement that there is no room for any considerable number of specialists in prosthodontia or orthodontia. He doesn't know of but a dozen or so. Of course one can't blame a man who spends his life in a small interior village for his myopic condition; but we are surprised that the versatile editor of the *Items of Interest* should not correct such a pessimistic statement. There have been specialists for generations in dentistry, in fact, it might not be difficult to make it seem that most practitioners specialize in practice to a considerable extent. It takes a broadly-educated and equipped man to practice "just dentistry" acceptably. The larger number probably specialize in filling teeth—and too many of them with amalgam—but there are hundreds of practitioners who give the greater part, if not all, of their time to the practice of some single department. We have extractors, orthodontists, plate-makers, crown and bridge-workers, inlay-workers, oral surgery and therapeutics in the treatment of pyorrhea and applied electricity and other departments of the healing art. The fact of the matter is that there are not many men who are making reputations for themselves in these days, who are not working diligently at some one thing. It looks very much as though we could not do better than to encourage specialization by every means in order that we may secure progress. The practice of orthodontia has never made such strides toward perfection of methods as in the last ten years, and there are more specialists in orthodontia to-day than ever before. And we may say that there is more room for thoroughly-equipped men in this department than anywhere else.

